# Multi-modality organ-based approach to expected imaging findings, complications and recurrent tumour in the genitourinary tract after radiotherapy

**DOI:** 10.1007/s13244-013-0295-z

**Published:** 2013-11-26

**Authors:** Nicola Schieda, Shawn Christopher Malone, Omran Al Dandan, Parvati Ramchandani, Evan S. Siegelman

**Affiliations:** 1 Department of Medical Imaging C1, The Ottawa Hospital, The University of Ottawa, 1053 Carling Avenue, Ottawa, Ontario Canada K1Y 4E9; 2 Ottawa Hospital Regional Cancer Center (Division of Radiation Oncology), The Ottawa Hospital, The University of Ottawa, 501 Smyth Road, Ottawa, Ontario Canada K1H 8L6; 3 Department of Medical Imaging C1, c/o Ms. Laura Lang, The Ottawa Hospital, The University of Ottawa, 1053 Carling Avenue, Ottawa, Ontario Canada K1Y 4E9; 4 Department of Radiology, The Hospital of the University of Pennsylvania, 3400 Spruce St, Philadelphia, PA 19104 USA

**Keywords:** Genitourinary tract, Imaging, Radiotherapy, Tumour

## Abstract

**Purpose:**

Radiotherapy (RT) is an integral component in the management of many abdominal and pelvic malignancies. Imaging follow-up in patients who have received RT is performed to assess for treatment response, evaluate for tumour recurrence and to diagnose complications related to treatment. The purpose of this pictorial review is to depict the expected imaging findings and potential complications following RT in the genitourinary (GU) tract using an organ-based approach and to review the diagnosis of locally recurrent tumour in the GU tract following RT.

**Conclusions:**

Some GU malignancies, namely cervical and prostatic carcinoma, can be treated with radical RT with intent to cure. More frequently, the GU tract is indirectly treated as a result of RT to adjacent cancers. Expected imaging findings, RT-related complications and the diagnosis of recurrent tumour following RT in the GU tract often necessitate a multi-modality imaging approach, the incorporation of functional imaging techniques and an organ-based approach for diagnosis.

## Introduction

Radiotherapy (RT) is commonly used for the treatment of various malignancies in the abdomen and pelvis. RT can be delivered using external (three-dimensional [3D] conformal, intensity modulated, stereotactic radiosurgery, proton therapy) or internal (brachytherapy) delivery systems which target the primary tumour/organ, attempting to minimise damage to adjacent structures. The genitourinary (GU) system is often involved when RT is administered in the abdomen and pelvis. GU involvement can be direct (if treatment is given for a primary GU malignancy) or indirect (when the primary target is in close proximity to adjacent organs of the GU tract). In the upper abdomen, RT is not the primary radical treatment option for the management of renal, adrenal or upper tract urothelial tumours [1–3]. These organs are commonly involved indirectly when RT is used to treat adjacent tumours in the spine, liver and pancreas for example. In the pelvis, the cervix and vulva in woman and the prostate gland in men, are frequently treated with radical RT [4, 5]. These organs, in addition to the urinary bladder, uterus and ovaries, are also involved indirectly during the treatment of other pelvic malignancies such as rectal cancer.

In the last decade there have there have been significant improvements in RT treatment and delivery techniques that should help to minimise the risk of early and late RT toxicity. Improvements in imaging allow radiation oncologists to better define tumour tissue versus organs at risk. Modern RT techniques including intensity modulated (IM) RT and proton beam RT help minimise doses to critical normal tissues. In addition, modern linear accelerators have cone-beam technology for daily image guided treatment delivery (image-guided [IG] RT). IGRT allows oncologists to use tight RT treatment margins around tumours helping minimise the risk of collateral normal tissue damage. Early recognition of RT toxicity is important for effective conservative and surgical management strategies. It is critical to distinguish radiation toxicity from recurrence as the treatment strategy and prognosis is quite different.

The purpose of this review is to depict the expected imaging findings following RT to the GU tract, using an organ-based approach. The importance of imaging for the diagnosis of RT-induced complications and recurrent tumour is also reviewed, emphasising the role of multi-modality imaging for diagnosis.

## Kidneys

In adults, RT is rarely given for the radical treatment of renal tumours, but may be given in the setting of salvage therapy or palliation [1]. In paediatric patients, RT is commonly used for the treatment of Wilms’ tumour [6, 7]. When RT is administered to the upper abdomen for other cancers, portions of the kidneys frequently also receive radiation dose. Radiation-induced renal injury (radiation nephropathy) was first recognised in 1906, although it only became well understood after a case series of men treated with RT for seminoma was reported in 1952 [8]. The kidney is a radiosensitive organ with a whole organ tolerance dose (5 % complication rate in 5 years) of 20 Gy [8]. The accepted threshold dose of irradiation that will cause radiation nephropathy is a total dose of 28 Gy, fractionated in 5 weeks or less [8]. Clinically, patients present with azotaemia, hypertension and anaemia, which can progress to renal failure[8]. In order to minimise the risk of radiation nephritis, advanced RT planning techniques, such as IMRT, can be used to decrease the volume of kidney receiving significant radiation dose.

**Fig. 1:**
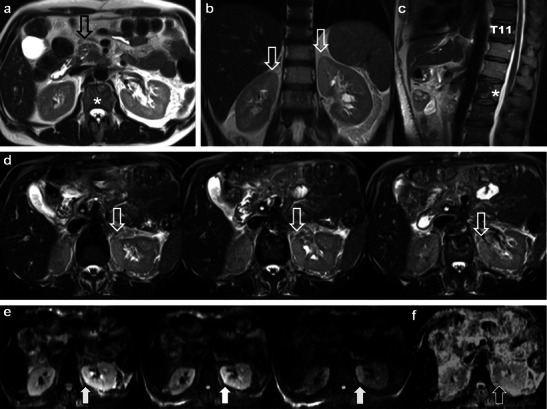
A 50-year-old man with pancreatic head cancer. Baseline axial T2 single-shot fast spin echo (ssFSE) pre-treatment MRI (**a**) demonstrates the primary tumour (*open black arrow*) and L2 metastasis (*asterisk*). The patient underwent Whipple’s procedure and external beam radiotherapy (RT) for L2 metastasis (30Gy over ten fractions). Follow-up MRI was performed 9 months after treatment. Coronal (**b**), sagittal (**c**) T2 ssFSE and axial fat suppressed T2 ssFSE (**d**) demonstrate increased T2 signal intensity in the medial upper pole renal cortices (*open white arrows* in **b** and **d**) consistent with acute radiation nephropathy. Note marrow replacement in the spine and partially treated L2 metastasis (*asterisk*) in **c**. Areas of radiation nephropathy demonstrate restricted diffusion in the medial upper poles (*solid white arrows*) on axial fat suppressed diffusion weighted echo planar (EPI) images (B50, B400 and B800—*left to right* in **e**). There is corresponding low signal intensity (*black arrow*) on ADC map (**f**)

**Fig. 2:**
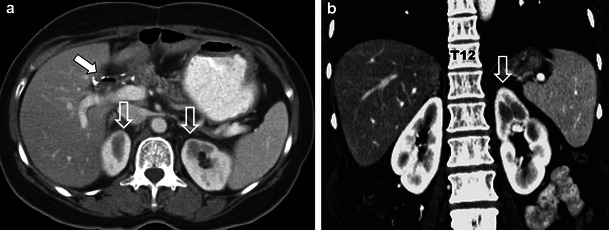
A 49-year-old woman who underwent pylorus sparing Whipple’s procedure and RT (5,940 cGy) for ampullary cholangiocarcinoma demonstrates typical findings of chronic radiation nephropathy on 3-year follow-up imaging. Axial (**a**) and coronal (**b**) contrast-enhanced CT (CECT) images demonstrate focal atrophy of the medial upper poles of both kidneys with a smooth contour (*open white arrows*). Note regional osteopenia in L1-L3 in (**b**) and post-operative pneumobilia in the bile duct (*solid white arrow* in **a**) indicating patent choledochojejunostomy (not shown)

The imaging findings of radiation nephropathy are well described. The kidneys may appear normal in acute radiation nephropathy [9]. In the acute setting, the kidneys may show decreased or absent function, resulting in a delayed or persistent nephrogram [10]. The imaging findings of radiation nephropathy have not, to our knowledge, been described using magnetic resonance imaging (MRI). Decreased T1 and increased T2 signal intensity in that portion of the affected kidney is commonly observed. These areas may also demonstrate restricted diffusion, which is of uncertain aetiology but may be on basis of cell death and cytotoxic oedema (Fig. 1). In the chronic setting, radiation nephropathy results in atrophy and scarring of the renal cortex with a smooth contour (Fig. 2) [10]; imaging findings indicative of irreversible damage. The proportion of patients with imaging findings of acute radiation nephropathy that progress to irreversible renal damage is, to our knowledge, unknown.

## Adrenal glands

**Fig. 3:**
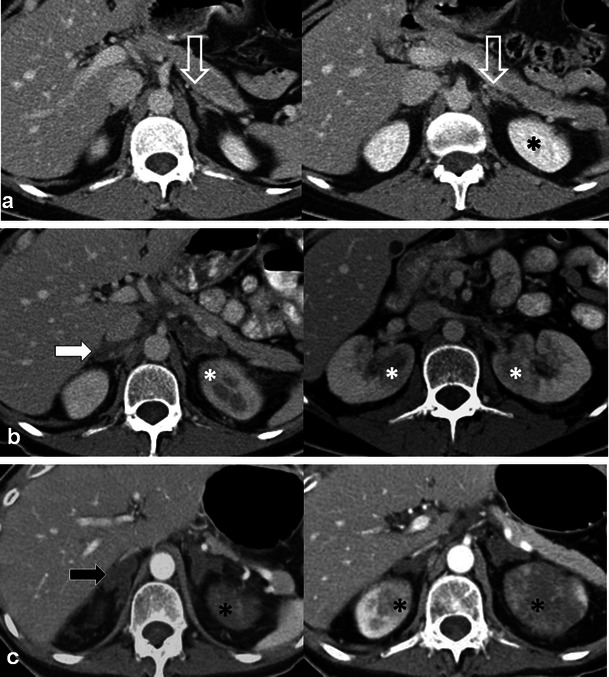
A 56-year-old man with metastatic rectal cancer to lungs and L3 thecal sac (not shown). Baseline axial CECT images (**a**) demonstrate normal adrenal glands and kidneys (*open white arrow* and *asterisk*). The patient received external beam RT to treat the thecal metastasis. Three-month follow-up axial CECT images (**b**) after therapy reveals delayed nephrogram in the upper pole of both kidneys (*asterisk*) typical findings of acute radiation nephropathy given the appropriate clinical history of prior RT. The patient also developed bilateral and symmetric low-density thickening of the adrenal glands (*white arrows*). The imaging findings were thought to represent acute radiation-induced adrenal injury. Final follow-up axial CECT images (**c**) performed 2 months after (**b**) reveal imaging findings compatible with worsening radiation-induced renal (*asterisk*) and adrenal (*black arrows*) injury, at which point the patient developed adrenal insufficiency

RT is rarely given for the salvage or palliative treatment of adrenal tumours, with surgery (for primary) and chemotherapy (for secondary) tumours representing the preferred treatment options [2]. RT can be given to treat or palliate high risk and aggressive neuroblastoma in children [11]. We previously reported the first case of acute adrenal injury following external beam RT of the spine [12], in which the patient developed adrenal insufficiency during the course of disease. The adrenal glands are reported to be radio-resistant, thought to be protected by the adrenal medulla and by their proximity to adjacent organs [13]. RT-induced adrenal injury may result in bilateral, symmetric, diffuse low-density thickening of the adrenal glands on contrast-enhanced computed tomography (CT) with co-existing imaging findings of radiation nephropathy (Fig. 3) [12]. The imaging findings of chronic radiation-induced adrenal injury are not known.

## Ureters

**Fig. 4:**
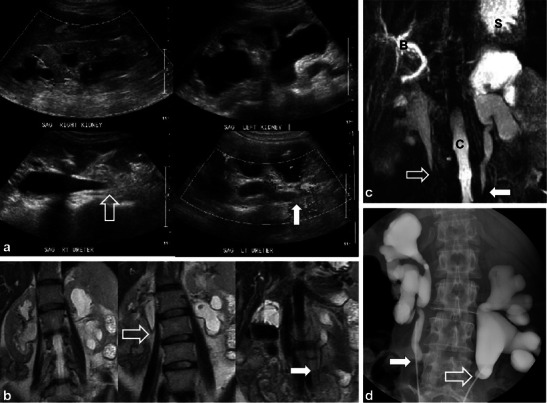
A 52-year-old woman with metastatic appendiceal mucinous adenocarcinoma treated with prior surgery and chemo-RT. Sagittal ultrasound images (**a**) show bilateral hydroureteronephrosis with smooth, tapered, transition of the right (*open white arrow*) and the left (*solid white arrow*) proximal ureters. Coronal T2 ssFSE (**b**) images confirm the presence of bilateral hydroureteronephrosis and smooth, tapered narrowing of both proximal ureters (*right and left*—*open and solid white arrows*). Coronal maximum intensity projection (MIP) image from heavily weighted T2 coronal 3D MRCP (**c**) also depicts the bilateral hydroureteronephrosis and the areas of transition in the proximal ureters (*open and solid white arrows*) (*B* common bile duct, *S* stomach, *C* spinal canal). Overhead images (**d**) from bilateral retrograde urethrogram (RUG) demonstrates correlation of findings between modalities with classic features of radiation induced ureteric strictures again noting smooth, tapered margins (*open and solid white arrows*) of both ureters

**Fig. 5:**
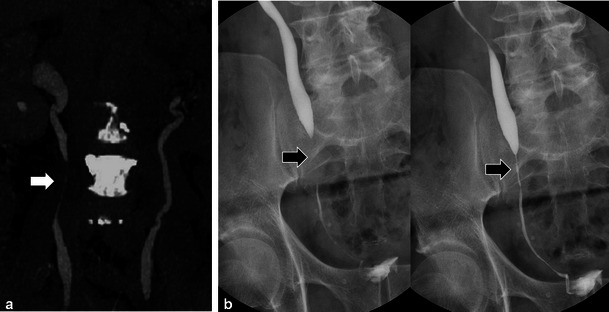
Coronal maximum intensity projection (MIP) from CECT urogram (**a**) of a 68-year-old man with diffuse metastatic prostate carcinoma (note sclerotic metastases in spine) treated with external beam RT for pelvic disease remotely. Moderate hydroureteronephrosis is present in the right collecting system with a smooth tapered long segment narrowing of the mid right ureter (*white arrow*) in keeping with a radiation induced ureteric stricture. Overhead AP radiographs from antegrade urogram (**b**) through indwelling nephrostomy tube (not shown) in a different 65-year-old man with previous history of colonic carcinoma treated with low anterior resection and pelvic RT depict a classic radiation induced stricture (*black arrows*) with smooth, tapered margins and long segment involvement just above the uretero-vesicle junction

RT is rarely given for upper tract urothelial tumours, but can be used in adjuvant or salvage therapy [3]. The ureters are relatively radio-resistant [9]. Ureteral strictures are uncommon complications of RT, occurring most commonly in patients treated for pelvic malignancy with reported frequencies between 1 and 3 % [9; 10]. Strictures can be focal but are usually long with characteristically smooth and tapered margins (Figs. 4 and 5) [9]. The most common location of radiation-induced ureteric stricture is at or just above the uretero-vesicular junction [14]. Strictures can occur acutely (within 6 months of RT) but are more commonly seen in the chronic setting with latency periods reported up to 10 years after treatment [9, 14]. In contrast to radiation-induced strictures, malignant strictures are irregular and tend to have abrupt and shouldered margins with an associated mass [14]. Other manifestations of ureteric injury following RT are rare and include fistula formation and reflux due to incompetence of the intramural portion of the ureter related to bladder fibrosis [15].

## Urinary bladder and urethra

The definitive treatment option for urinary bladder tumours is surgery. A recent review concluded that RT for superficial tumours and in the routine pre/post-operative setting is not indicated [16]. Combined RT and chemotherapy is an option for selected cases of bladder cancer in patients opting for bladder preservation. RT alone should only be considered for palliation or in non-operative patients who cannot also receive chemotherapy [16].

Radiation-induced bladder injury is often related to RT of other pelvic malignancies. The bladder is the most radiosensitive organ in the urinary tract [9]with the incidence of radiation cystitis ranging from 3 % to 12 %, depending on total dose and volume of bladder treated [15]. Doses of 30 Gy over 3–4 weeks can result in mild injury with doses of 60–70 Gy, resulting in more severe injury and possible long-term complications [9]. Radiation cystitis is often separated into an acute phase (<3–6 months), where pathologically there is oedema and hyperaemia of the mucosa and submucosa with lymphocytic infiltration and a chronic phase (>6 months) where there is fibrosis, mucosal atrophy, radiation telangiectasia and rarely fistula formation [9].

**Fig. 6:**
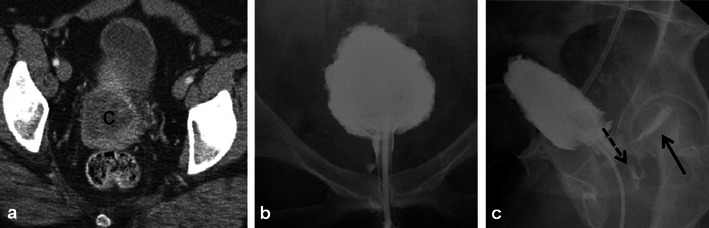
A 49-year-old woman post RT for locally advanced cervical squamous cell carcinoma depicted on pre-treatment axial CECT (**a**) which demonstrates the large necrotic cervical tumour (*C*). Following RT, a conventional cystogram was performed to evaluate for suspected fistula. Late filling AP (**b**) and oblique (**c**) radiographs of the urinary bladder demonstrate classic findings of radiation cystitis with a reduced capacity bladder that is elevated from the pelvic floor and has a rounded appearance. Note bladder wall thickening and trabeculation. Oblique radiograph (**c**) also depicts filling of the vagina (*dashed black arrow*) and rectum (*solid black arrow*) in keeping with complex radiation-induced vesicovaginal and rectovaginal fistulas

**Fig. 7:**
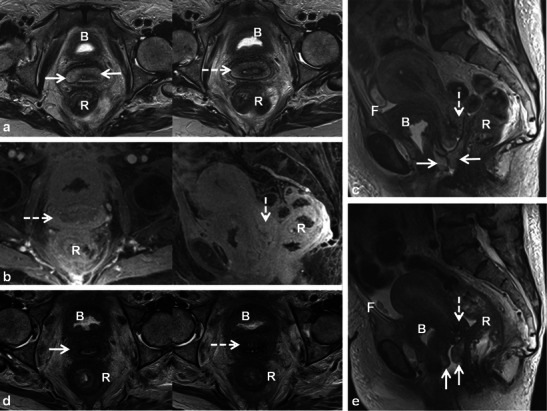
A 69-year-old woman with bladder adenocarcinoma status post transurethral resection receiving adjuvant pelvic external beam RT. Routine follow-up pelvic MRI demonstrates widening of the cervix and thickening of the anterior and posterior vaginal fornices with increased T2 signal intensity on axial and sagittal T2 TSE (*dashed and solid white arrows* respectively in **a** and **c**). Also note diffuse thickening and increased T2 signal in the rectum (*R*). Axial and sagittal T1 fat-saturated gradient echo images (**c**) after gadolinium injection also depict the findings (*dashed arrows*, *R*). Note pelvic free fluid (*F*), stranding and oedema in the pelvis. Follow-up pelvic MRI obtained 3 months later show similar but improving findings in the cervix (*dashed arrows*) and vagina (*solid arrows*) on axial (**d**) and sagittal (**e**) T2 TSE sequences consistent with acute radiation cervicitis and vaginitis. Note worsening radiation proctitis (*R*) with increasing pelvic free fluid (*F*). Bladder wall thickening and increased T2 signal (*B*) was felt to be due to radiation cystitis in this patient post resection, although residual or recurrent tumour could have a similar appearance

Fluoroscopic images demonstrate reduced capacity, elevation of the bladder from the pelvic floor, variable degrees of wall thickening and a characteristic rounded appearance (Fig. 6) [9, 10]. Calcifications of the bladder wall may be seen [9]. CT shows similar findings with bladder wall thickening and decreased size [9]. Peri-vesical stranding (in the acute setting) and increased fatty deposition (in the chronic setting) may be seen. With haemorrhagic cystitis, high density can be seen in the bladder lumen [9]. Imaging findings with MRI are similar, although early changes of RT-induced cystitis are better depicted with T2-weighted sequences, which demonstrate variable degrees of increased signal in the bladder wall depending on the severity of the injury [9, 14]. Mild cystitis affects primarily the mucosa and submucosa, and severe toxicity results in transmural involvement (Fig. 7) [9]. MRI can demonstrate preferential involvement of the posterior bladder wall and trigone, with later involvement of the entire bladder [14]. Increased mucosal enhancement may be observed with contrast-enhanced CT or MRI [14]. A severe complication of radiation cystitis is development of fistula to the bowel or reproductive organs, which is discussed later. Differentiation of residual or recurrent tumour from post-RT changes in the bladder is difficult and imaging findings, to our knowledge, have not been described. If there is clinical concern in this setting, cystoscopy is likely indicated for tissue sampling.

**Fig. 8:**
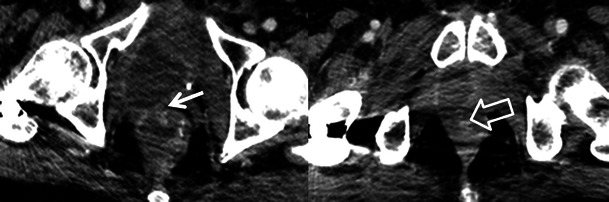
A 63-year-old man with prostatic carcinoma, originally treated with brachytherapy followed by salvage external RT for locally recurrent tumour. Consecutive CECT axial images (**a** and **b**) demonstrate a complex fistula between the rectum and urinary bladder (**a**) and more inferiorly between the rectum and the urethra (**b**) from prior RT. Initial management for this patient consisted of urinary diversion with bilateral nephrostomy tubes (not shown). Surgery was subsequently attempted, but after total prostatectomy was performed, the complex fistulous disease could not be repaired

Radiation-induced urethral injury is typically related to brachytherapy treatment of prostatic carcinoma [17]. Brachytherapy complications include urethral strictures and, rarely, urorectal fistulas [17]. Urethral strictures can develop in up to 5 % of patients treated with brachytherapy and are dependent on dose. Urethral strictures involve the posterior membranous urethra and are treated with dilation/incision [17]. Urorectal fistulas (Fig. 8) are rare serious complications. Fistulas are managed initially with urinary diversion and later in some patients primary repair can be attempted [18].

## Uterus, cervix, ovaries and vulva

RT for gynaecological malignancies varies by site and stage; however, it is most commonly used for the management of locally advanced cervical [4] and vulvar [5] cancers and less commonly used in the treatment of other uterine and ovarian malignancies [4, 5, 19].

**Fig. 9:**
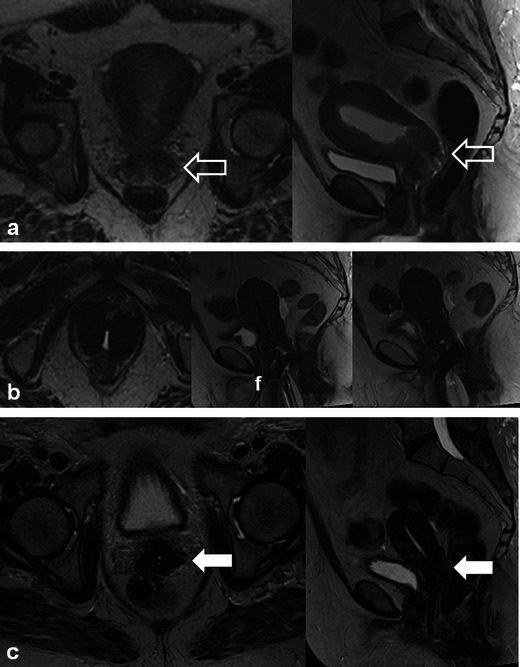
A 60-year-old woman with cervical squamous cell carcinoma, treated with brachytherapy, demonstrating complete response to treatment. Initial staging pelvic MRI depicts the fungating cervical mass with left parametrial invasion (*open white arrows*) on coronal oblique and sagittal T2 TSE (**a**). Also note the mass is causing malignant hydrometra due to cervical obstruction. Follow-up pelvic MRI for treatment planning depicts optimal positioning of ovoid and tandem intracavitary brachytherapy device on axial and sagittal T2 TSE with intracavitary device at the level of the cervical tumour and probe tip in the endometrial cavity. Foley catheter (**f**) in situ. Follow-up pelvic MRI performed 1 year after RT demonstrates complete response with reconstitution of the normal zonal anatomy of the cervix and presence of homogeneous low signal intensity of the cervical stroma (*white arrows*) on axial and sagittal T2 TSE (**c**), which are reliable indicators of complete response and absence of tumour in the post-irradiation cervix. Low T2 signal has replaced intermediate signal in the left parametrium (*white arrow* on axial image in **c**) indicating parametrial fibrosis

**Fig. 10:**
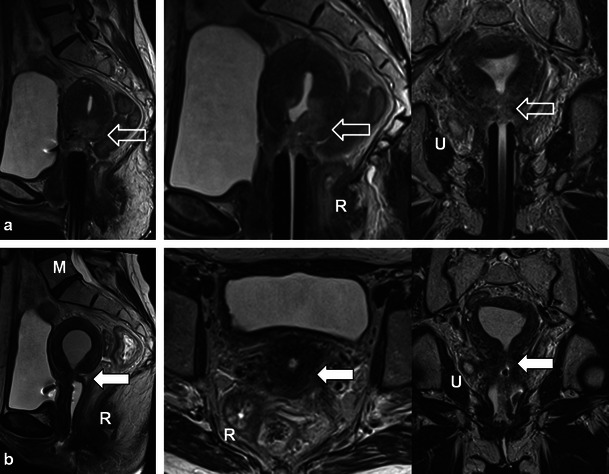
A 46-year-old woman with locally advanced (Stage IIIB) cervical squamous cell carcinoma (*open white arrows*) with right ureteral involvement (*U*) depicted on treatment planning pelvic MRI with sagittal and coronal oblique T2 TSE sequences. Note the tumour is of intermediate T2 signal intensity (*open white arrows*) and that the brachytherapy device is appropriately positioned at the level of the tumour. Follow-up MRI demonstrates interval reduction of tumour size and T2 signal intensity with focal atrophy at the cervical os (*closed white arrow*) with progression of now moderate hydrometra on sagittal, axial and coronal oblique T2 TSE sequences. The findings are consistent with treatment response and post therapeutic cervical stenosis which is a common finding after RT for cervical carcinoma. Also note radiation proctitis (*R*) in (**a**) which resolves after therapy in (**b**) and fatty marrow replacement (*M*) in **b** after therapy

**Fig. 11:**
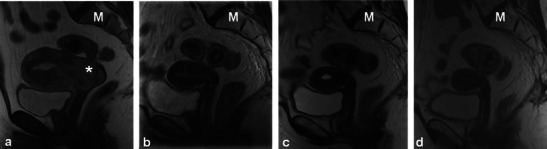
A 42-year-old woman with cervical squamous cell carcinoma showing expected post-RT findings over consecutive MRI examinations. Sagittal T2 TSE sequences performed at baseline (**a**) and 1-year intervals thereafter (**b**,**c** and **d**). Baseline imaging **a** demonstrates extensive tumour centred in the cervix with uterine and vaginal invasion (*asterisk*), and malignant obstruction of the cervical os causing hydrometra. Parametrial invasion was also present (not shown). Note normal marrow signal intensity (*M*). One-year follow-up imaging **b** demonstrates reconstitution of the normal zonal anatomy of the cervix and presence of homogeneous low signal intensity of the cervical stroma reliable indicators of complete response. Note global uterine atrophy and fatty replacement of the pelvic marrow, which are expected imaging findings following pelvic RT. Subsequent follow-up examinations performed 2 (**c**) and 3 (**d**) years after therapy show progressive uterine atrophy but no recurrent tumour

Post-RT findings in the female reproductive organs and pelvis are non-specific. In the early post-RT setting, increased T2 signal intensity with MRI may be seen in the cervix and vagina due to inflammatory cervicitis and vaginitis (Fig. 7). These findings can occur in conjunction with other findings of acute radiation injury in other pelvic organs such as the urinary bladder and bowel (Fig. 7). Other non-specific findings seen after acute pelvic RT include inflammatory stranding, oedema and free fluid in the pelvis. In the chronic setting, the uterus, cervix and ovaries atrophy. Low signal intensity within these organs on T2-weighted sequences is commonly observed (Figs. 9, 10 and 11) [20]. Thickening (without nodularity) and low T2 signal of the uterosacral ligaments and pelvic sidewall are also common [20]. In younger patients the ovaries may be intentionally transposed out of the pelvis prior to RT to preserve fertility. The normal ovaries in this instance should not be mistaken for lymphadenopathy or peritoneal implants.

Of the gynaecological malignancies, cervical carcinoma is most commonly treated with RT. Patients with Stage IB or IIA tumour may be treated with surgery or RT [20], while patients with advanced disease (Stages IB, III and IVA) are usually treated with RT combined with chemotherapy [4]. Optimal cure rates are achieved by combined external beam and intracavitary RT [21]. Brachytherapy devices vary in shape and configuration, but the applicator and the intracavitary component should always be positioned at the level of the tumour within the cervix (Figs. 9 and 10) [21]. Probe tip, if present, should pass through the cervix into the endometrial cavity (Fig. 9) [21]. Interstitial brachytherapy needles are used in conjunction with the intracavitary device in selected cases of cervical cancer [21]. Intracavitary brachytherapy devices are MRI compatible and are best evaluated on fast/turbo spin echo sequences which minimise susceptibility artefacts that can render the use of gradient echo sequences prohibitive. Complications from brachytherapy placement are common (up to 8 %) and are mostly related to perforation (usually at the uterine fundus and posterior vaginal fornix) [21]. Direct trauma to adjacent bowel from perforation is uncommon but increased local dose can lead to long term fistula and stricture formation [21].

Patients undergoing RT for the treatment of cervical cancer are not commonly imaged in the early post-RT setting [20]; however, a widened endocervical canal and non-specific increased T2 signal in the cervical stroma and vagina may be observed [20]. An early (<3 months) decrease in signal intensity and tumour volume indicates a favourable response to treatment [4]. Recently, using diffusion-weighted imaging (DWI), an increase in the apparent diffusion coefficient (ADC) value of the tumour has been described with RT response [22]. Later, there is an expected linear decrease in uterus and cervical volume as well as T2 signal intensity (Figs. 9, 10 and 11) [4]. Areas of parametrial invasion will be replaced by low T2 signal intensity fibrosis (Fig. 9) [4]. Reconstitution of the normal cervical zonal anatomy with homogeneous low signal intensity of the cervical stroma is a reliable indicator of complete response and absence of tumour in the post-irradiation cervix (Figs. 9 and 11) [4]. Positron emission tomography (PET)-CT has been described as early as 2–4 weeks after chemo-RT for the assessment of treatment response, but is typically performed after at least 3 months following the initiation of therapy [23].

**Fig. 12:**
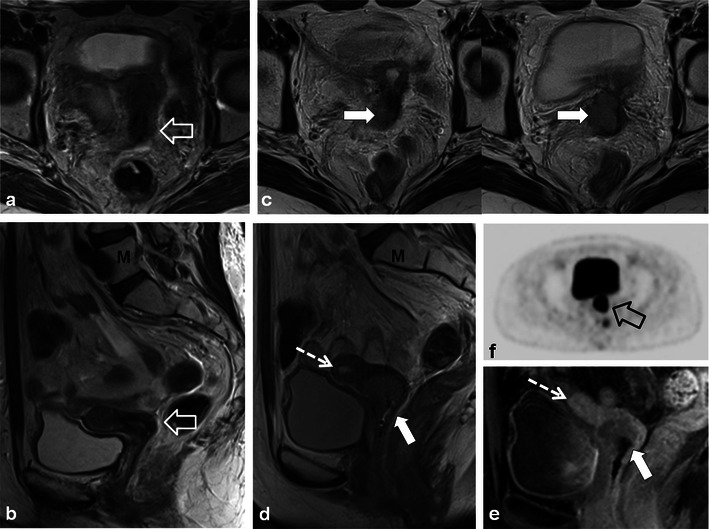
A 56-year-old woman with previous cervical squamous cell carcinoma treated remotely with RT. Post-therapy baseline pelvic MRI demonstrates the normal cervical zonal anatomy with homogeneous low T2 signal intensity (*open white arrows*) on axial and sagittal T2 TSE (**a** and **b**) consistent with complete response and a tumour-free irradiated cervix. Note global uterine atrophy in **b** commonly seen following pelvic RT. Follow-up pelvic MRI performed 1 year later demonstrates interval development of a well-defined intermediate T2 signal intensity mass in the cervix (*closed white arrow*) on axial (**c**) and sagittal (**d**) T2 TSE. Note interval development of hydrometra (*dashed arrow* in **d**) due to the obstructing mass. The mass is characteristically hypovascular in relation to the atrophic uterus after gadolinium injection on sagittal T1 fat saturated GRE image (**e**). Axial PET image (**f**) from whole-body ^18^F-fluorodeoxyglucose (FDG) PET study performed the same day shows corresponding area of avid FDG uptake. The imaging findings are consistent with locally recurrent tumour, which was proven at biopsy

**Fig. 13:**
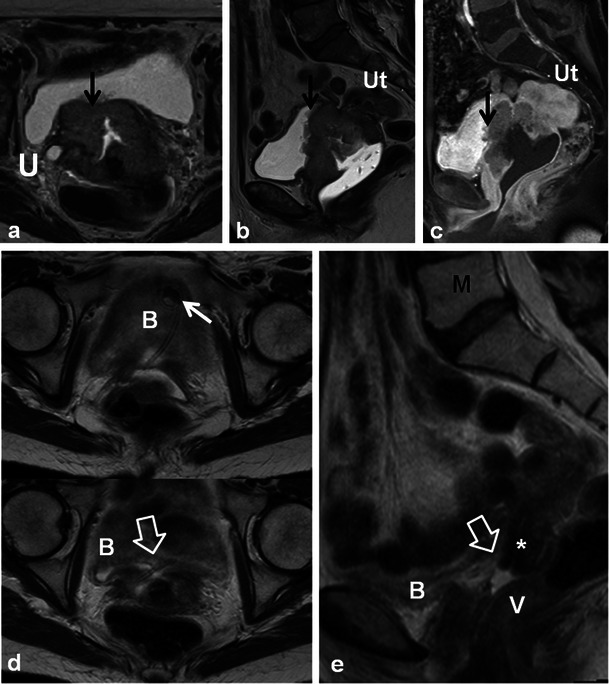
A 57-year-old woman with locally advanced (Stage IVa) squamous cell carcinoma of the cervix demonstrating invasion of the posterior wall of the urinary bladder (*black arrow*) and right ureter (*U*) at baseline MRI on axial and sagittal T2 TSE (**a** and **b**) and sagittal T1 fat-saturated post-gadolinium (**c**) gradient recalled echo (GRE) images. Note sterile ultrasound gel within vagina and atrophic uterus (*Ut*). Following RT there is a small but definite communication between the anterior wall of the vagina and the posterior wall of the urinary bladder (*open arrows*) depicted on axial (**d**) and sagittal (**e**) T2 TSE findings consistent with post-RT vesicovaginal fistula. A double-J ureteric stent (*short white arrow*) to relieve right hydroureteronephrosis and fatty marrow replacement (*M*) are also noted

**Fig. 14:**
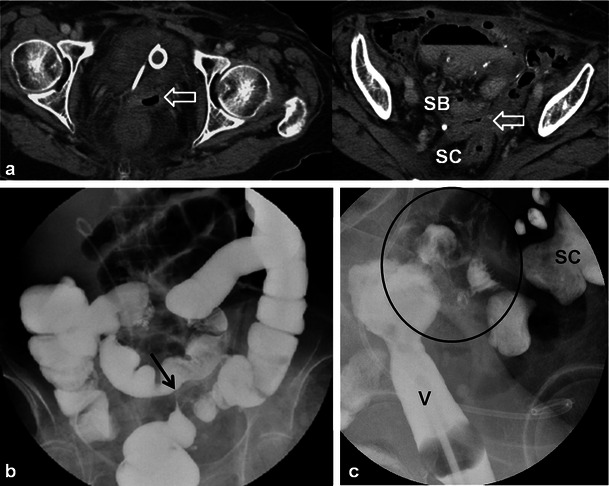
An 80-year-old woman with a history of endometrial carcinoma and ovarian carcinoma treated remotely with pelvic surgery and RT presenting with clinical symptoms of vaginal-enteric fistula. Initial CECT axial images (**a**) depict abnormal gas within the thickened vagina (*open white arrow*) which is in close proximity to both pelvic small bowel loops (*SB*) and sigmoid colon (*SC*). A colovesical fistula was suspected. Barium enema was subsequently performed (**b**) to confirm the findings. Overhead AP image from single contrast barium enema fails to demonstrate communication between the colon and vagina. Note short segment persistent narrowing of the sigmoid colon (*black arrow*) with tapered margins and preserved mucosal folds (not shown) consistent with radiation induced stricture. Direct vaginography was suggested for further evaluation. Oblique radiograph from direct vaginogram depicts maximal distention of the vagina (*V*) and residual barium in the sigmoid colon (*SC*) with uncomplicated diverticulosis. There is abnormal filling of pelvic small bowel loops (*black circle*). The findings were in keeping with fistula between the distal ileum and vagina

Cervical stenosis following RT is common (Fig. 10) [20]. It is important to differentiate benign hydrometra due to RT-induced cervical stenosis from locally recurrent tumour, which can also cause obstruction at the cervical os (Fig. 12). Absence of an obstructing mass and lack of intermediate T2 signal intensity are reliable differentiating features. Functional imaging techniques such as DWI and dynamic contrast enhancement (discussed later) may also increase diagnostic accuracy in this setting. Other complications associated with RT of cervical carcinoma (and pelvic RT in general) include ureteral stenosis, radiation enteritis/colitis and development of fistulous disease to the urinary bladder and bowel [4, 20]. Fistulous disease can occur decades after pelvic RT and often necessitates a multi-modality approach for diagnosis (Figs. 6, 13 and 14) [20]. RT-induced fistula can be difficult to differentiate from malignant fistulous disease caused by locally recurrent tumour and tissue sampling is often required for accurate diagnosis [4, 24, 25].

**Fig. 15:**
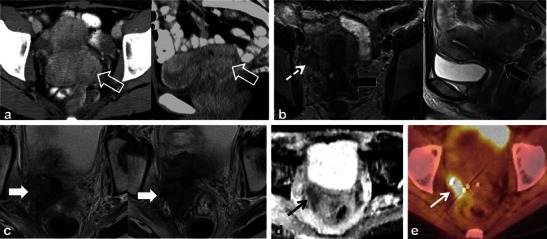
A 34-year-old woman with locally advanced cervical squamous cell carcinoma. Baseline pre-treatment axial and sagittal CECT (**a**) images demonstrate a large cervical tumour (*open white arrows*). Post-RT follow-up pelvic MRI performed 8 months later (**b**) depicts imaging findings consistent with complete response with reconstitution of the normal cervical zonal anatomy and homogeneous low T2 signal (*wide black arrows*) on axial and sagittal T2 TSE sequences. Note the small size and homogeneous low T2 signal without follicular activity in the right ovary (*dashed white arrow*) an expected post-RT finding. Follow-up pelvic MRI was performed 7 months later, axial T2 TSE images (**c**) reveal a heterogeneous ill-defined mass in the right parametrium that extends to the right pelvic side wall with areas of both intermediate and low T2 signal intensity which is suspicious for locally recurrent tumour (*solid white arrows*). In the early post RT setting immature fibrosis could have a similar appearance. DWI performed at same imaging session demonstrates restricted diffusion within this mass as low signal intensity on ADC map (*black arrow* in **d**). Subsequently whole-body PET-CT was performed (**e**) for the assessment of systemic disease, which depicts avid FDG uptake in the mass on fused axial FDG PET-CT image (*white arrow* in **e**). Biopsy was performed which confirmed recurrent tumour in this case involving both the central and lateral pelvic compartments

Patients treated with RT for cervical cancer are often followed clinically and with imaging to detect recurrences. Recurrent cervical cancer is defined as local tumour re-growth or the development of distant metastases at least 6 months after the lesion has regressed [4, 25]. Most relapses occur in the first 2 years after treatment [24]. Thirty percent of patients treated with radical RT will die from recurrent disease [4, 26]. Risk factors for recurrence include histological features, depth of invasion and nodal status [4, 26]. The most frequent sites of recurrence in the pelvis are in the central (cervix, uterus, vagina, parametria, ovaries, bladder or rectum) or lateral (pelvic side wall) compartments [4, 24–27]. After RT, 70 % of recurrences occur in the treated cervix [28]. Recurrences can also occur systemically in lymph nodes or visceral organs and bone. Imaging diagnosis of locally recurrent tumour varies by institution but is primarily performed with MRI and PET-CT [24]. Whole-body DWI is an emerging diagnostic tool which could be applied for the detection of systemic or locally recurrent tumour in cervical carcinoma [29]. The sensitivity of detecting pelvic recurrence is 90 % in both MRI and PET-CT with superior depiction of extra pelvic disease using PET-CT [28, 30]. Central compartment recurrence may be recognised on CT as soft tissue masses with variable necrosis or cystic change [4, 26, 27]. Evaluation of locally recurrent tumour is better with MRI when compared with CT [4, 24, 27]. Recurrent tumour demonstrates intermediate T2 signal intensity with MRI (Figs. 12 and 15) [4, 20, 27]. Interval change over serial examinations with development of intermediate T2 signal is the most reliable finding of locally recurrent tumour [28]. Within 6 months of therapy increased T2 signal intensity in the cervix can be seen from RT induced inflammation (Figs. 7 and 10) [4, 24]. Recurrent tumour enhances heterogeneously with variable degrees of necrosis (Fig. 12) [4, 20, 26, 27]. PET-CT reveals a heterogeneous mass in the central or lateral compartments with increased FDG uptake (Figs. 12 and 15) [28, 30].

Distinguishing between post-RT fibrosis and locally recurrent tumour can be challenging (Fig. 15) [4]. Dynamic-contrast enhanced (DCE) MRI and DWI are functional sequences that improve specificity [20, 24, 28, 31]. Recurrent tumour enhances earlier and to a greater extent than fibrosis [28, 32]. Recurrent tumour may demonstrate restricted diffusion, whereas radiation fibrosis often does not [28]. PET-CT is typically also performed in this setting, showing uptake in areas of recurrent tumour [30].

## Prostate, seminal vesicles and testicles

RT has no role in the primary treatment of non-seminomatous testicular tumours. Adjuvant retroperitoneal RT in seminoma is associated with increased risk of late second malignancies. Because of this risk, Stage I seminoma patients are offered active surveillance and radical RT is reserved for Stage 2 disease and for patients who relapse on surveillance [33]. Testicular atrophy may be seen following pelvic RT.

**Fig. 16:**
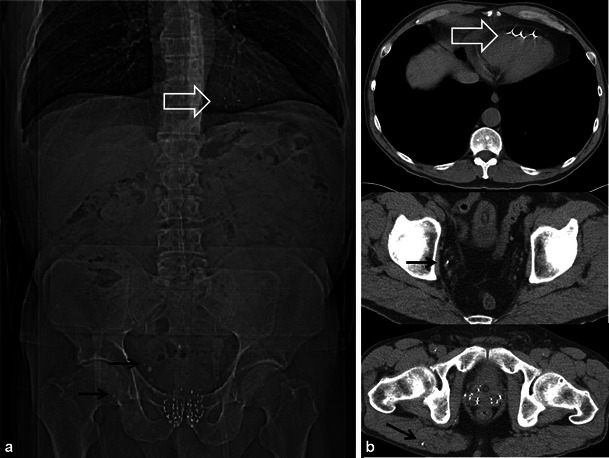
A 64-year-old man with prostate carcinoma treated with brachytherapy. Routine follow-up unenhanced CT reveals multiple radio-dense seeds in the prostate and several which have migrated out of the prostate revealed both on the scout tomogram (**a**) and axial CT images (**b**). Seeds are located in the right gluteus maximus and right pelvic veins (*black arrows*) and embedded in the right ventricle (*open white arrows*). Seed migration is common, occurring in up to 25 % of patients and should be noted on follow-up imaging

The treatment of prostate carcinoma varies depending on patient age, health status, clinical stage, prostate serum antigen (PSA) level and Gleason score at biopsy. Many patients are treated with RT. Internal RT (brachytherapy) is typically used in patients with low-risk prostate cancer and requires the transperineal insertion of radioactive seeds into the prostate gland. Post-procedural imaging will depict the seeds as radiodensities using radiography/CT and as areas of susceptibility artefact with MRI. Seed migration is common, occurring in up to 25 % of patients with migration sites such as the lungs, pelvis and mediastinum most often involved [34, 35]. Movement of seeds may result in inadequate dosimetry and possible morbidity in distant organs. Less common sites of migration include the liver, inguinal region and heart (Fig. 16) [34, 35]. Image-guided external RT techniques require the placement of fiducial markers (gold or platinum seeds) or other guidance systems in the prostate gland.

**Fig. 17:**
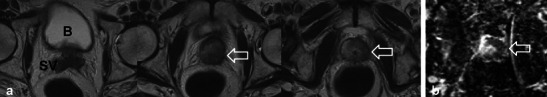
A 75-year-old man with prostate carcinoma treated remotely with external beam RT, presenting with biochemical relapse. Pelvic MRI was performed using phased array surface coils. Axial T2 TSE images (**a**) demonstrate typical findings following RT in the pelvis with low T2 signal and atrophy of both the prostate and small seminal vesicles (*SV*) when compared with pre-treatment imaging (not shown). Also note the poor differentiation of the prostate zonal anatomy. There is an amorphous area of lower T2 signal intensity diffusely in the left gland (*open white arrow*). DWI with calculation of ADC map (**b**) reveals a corresponding area of markedly restricted diffusion throughout the left gland (*open white arrow*) that represents biopsy proven recurrent tumour in this patient

**Fig. 18:**
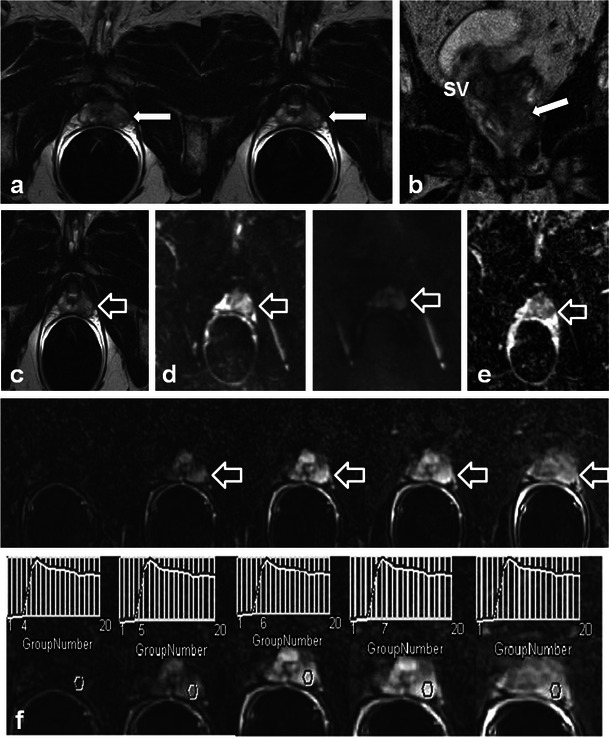
A 68-year-old man with prior external RT and biochemical failure with subsequent transrectal ultrasound guided biopsy demonstrating Gleason score 8 recurrent tumour in the left mid and basal peripheral zone. Multi-parametric endorectal coil prostate MRI depicts atrophic prostate gland and seminal vesicles with low T2 signal intensity and poor delineation of prostate zonal anatomy on axial and coronal T2 TSE images (**a**, **b** and **c**) which are typical post-RT changes. There is an intermediate T2 signal intensity area in the left mid and basal peripheral zone (*white arrows*), which is bulging the prostate capsule. Diffusion-weighted images (**d**) with B0 and B1,000 and ADC map **e** demonstrate restricted diffusion in this location (*open white arrows*). Corresponding axial pre- and selected post-dynamic-contrast-enhanced GRE images with semi-quantitative contrast curves demonstrate corresponding early rapid enhancement with steep upslope (*open white arrows* in **f**) and contrast washout in the area of known recurrent tumour

Morphological changes in the prostate and seminal vesicles are observed after both external RT and brachytherapy. The prostate gland and seminal vesicle atrophy and diffuse low signal intensity on T2-weighted sequences is observed (Figs. 17 and 18) [36]. Loss of the normal zonal anatomy due to chronic inflammation, glandular atrophy and fibrosis is common [36]. The normal peripheral zone glandular tissue actively secretes citrate and sparingly uses choline for cell membrane synthesis, resulting in high citrate and low choline levels [36]. In the irradiated peripheral zone, glandular tissue metabolism converts to citrate oxidising and utilises more choline for cell membrane synthesis. An increased choline + creatinine/citrate ratio is therefore observed in the normal irradiated prostate using MR spectroscopy [36]. The normal peripheral zone is composed of large amounts of glandular tissue with free diffusion of water molecules resulting in high apparent diffusion coefficient (ADC) values using DWI [36]. In the irradiated peripheral zone, ADC values decrease due to destroyed glandular function and reduced secretions in the acute setting and fibrosis in the chronic setting [37]. Treatment changes are also depicted within the primary tumour when imaged with serial MRI examinations with an expected decrease in T2 and an increase in ADC values [38]. Complications of pelvic RT for the treatment of prostate cancer are similar to pelvic RT complications in general and discussed earlier, but include mainly radiation cystitis, urethral or urinary bladder fistula formation and radiation proctitis.

The diagnosis of recurrent prostate cancer is based mainly on PSA kinetics [36]. Biochemical relapse or failure after RT is defined according to the Houston criterion, which is a nadir PSA level + 2 ng/ml within 5 years of completing therapy [36, 37]. Biochemical relapse can be on the basis of local recurrence or metastatic disease. Systemic recurrences are typically to lymph nodes and bone, and diagnosis can be achieved with CT and bone scan [39]. Whole-body DWI has also been described for the diagnosis of systemic disease in prostatic carcinoma [29]. ^18^F-Fluorocholine PET-CT or PET-MR is a variably available imaging modality that can also be used in the setting of biochemical relapse, mostly for the detection of systemic disease if salvage therapy for local recurrences is contemplated [40]. Approximately 30 % of patients treated with RT will suffer biochemical failure and this will typically occur at the site of the primary tumour [37, 41]. Intermediate T2 signal intensity at the site of the prior tumour may represent local recurrence. T2-weighted imaging alone lacks specificity and is insufficient to reliably diagnose local recurrence [36, 37, 41–43]. Multi-parametric MRI (two or more functional imaging tests) is better than T2-weighted imaging alone for the diagnosis of local recurrence [36, 37, 41–43]. Comparison of the three functional imaging tests (DWI, DCE and MR spectroscopy ) in the diagnosis of recurrent prostate cancer is beyond the scope of this manuscript, but has been described in detail elsewhere. Locally recurrent tumour shows more restricted diffusion (lower ADC values) with DWI, earlier onset and greater magnitude of enhancement with rapid washout of contrast material as well as larger quantitative perfusion (K*trans*, K*ep* and v*e)* parameters with DCE and higher choline + citrate/creatinine ratios using MR spectroscopy when compared with the normal post-irradiated peripheral zone (Figs. 17 and 18) [36, 37, 41–43].

## Conclusion

In conclusion, imaging changes following RT treatment in the GU tract are common and should be recognised in the post-RT setting. RT related complications in the GU tract are variant and often necessitate a multi-modality approach for diagnosis. The diagnosis of locally recurrent tumour after RT is complex and often requires the application of advanced functional imaging techniques for diagnosis.
